# Summary of a Workshop on the Development of Health Models and Scenarios: Strategies for the Future

**DOI:** 10.1289/ehp.7380

**Published:** 2004-12-07

**Authors:** Kristie L. Ebi, Janet L. Gamble

**Affiliations:** ^1^Exponent Health Group, Alexandria, Virginia, USA; ^2^Global Change Research Program, Office of Research and Development, U.S. Environmental Protection Agency, Washington, DC, USA

**Keywords:** climate change, health models, health scenario development, policy

## Abstract

A workshop was convened in July 2003 by the Global Change Research Program, Office of Research and Development at the U.S. Environmental Protection Agency, to review current strategies for developing human health models and scenarios in the context of global environmental change, particularly global climate change, and to outline a research agenda that effectively characterizes the interplay of global change with the health of human populations. The research agenda developed at the workshop focused on three issues: *a*) the development of health models, *b*) the development of health scenarios, and *c*) the use of health models and health scenarios to inform policy. The agenda identified research gaps as well as barriers to the development and use of models and scenarios. This report summarizes the workshop findings.

There is growing interest in modeling the potential health impacts of global environmental changes and in exploring, through scenario development, how current health risks could evolve with changes in environmental, technologic, economic, and societal conditions. Developing health scenarios can provide important insights into the complex relationships between humans and their environment and thus inform policy approaches to sustainable development, including intergenerational equity. Accordingly, the Global Change Research Program in the Office of Research and Development at the U.S. Environmental Protection Agency convened a workshop on 21–22 July 2003 in Washington, DC, to develop a research agenda for the development of health models and scenarios that characterize the interplay of global environmental change, particularly global climate change, with the health of human populations. Workshop participants had expertise in public health, climate change, modeling, scenario development, and policy development.

Models describe, quantitatively and/or qualitatively, relationships among various drivers of human health outcomes. For example, models can project disease burdens when input parameters change, such as the potential health consequences of changes in prevailing weather patterns. Scenarios, on the other hand, do not project whether a particular event, such as a disease outbreak, might occur. Scenarios paint pictures of possible or plausible futures and explore the different outcomes that might result when current conditions change (e.g., future socioeconomic and technologic developments, developments in medical care, population demographics, policy interventions). There is a strong interplay between models and scenarios. Scenarios can be used as inputs into models to project changes in the intensity and range of climate sensitive diseases, and models often underlie scenarios. For example, the Standardized Reference Emission Scenarios incorporate linked models of human economic activity and their resulting anthropogenic emissions with models of the earth system responses to the forcings from these emissions (Nakicenovic et al. 2000). Health models have been coupled with these scenarios to project disease burdens under different assumptions (e.g., Campbell-Lendrum et al. 2003; Hayhoe et al. 2004; Van Lieshout et al. 2004).

Both models and scenarios are needed to further our understanding of the potential impacts of climate variability and change on human health and well-being. A better understanding of the potential impacts can facilitate the development and implementation of effective and efficient adaptations that reduce negative impacts and take advantage of any opportunities that arise. In addition, this understanding can inform policy-relevant analyses of the possible consequences of mitigation policies. This is particularly critical because past scenario studies did not adequately incorporate population health issues.

Using models to project the potential health impacts of climate change poses a difficult challenge. In addition to affecting the intensity and range of climate sensitive diseases, global climate change may influence major risk factors for adverse health outcomes such as per capita income, nutrition, access to clean water, local pollution control, and large-scale migrations (McMichael et al. 2001). Another challenge to model projections arises because the sensitivity and adaptive capacity of exposed populations vary considerably depending on factors such as population density, level of economic and technologic development, local environmental conditions, preexisting health status, and the quality and availability of health care and public health infrastructure (Woodward et al. 1998). Projections of current trends into the future should not assume a future that looks like the past. Although the nature and extent of the potential health impacts of global environmental change are inherently uncertain, models can be used to explore the range of possible health burdens that could be faced by a population under a particular set of assumptions.

Scenarios are useful tools to aid assessments of the potential future impacts of global climate change. Because scenarios focus on understanding potential vulnerabilities and adaptive responses, they allow a deeper understanding of the potential health risks associated with climate and other global changes. More important, scenarios and the assessments they inform act as a bridge between science and policy. They can influence policy making by summarizing and synthesizing scientific knowledge in a form that helps policy makers visualize the dimensions of an issue and devise policies and processes to address risks and to increase future adaptive capacity (Scheraga et al. 2003).

There are many definitions of scenarios. The workshop focused on the definition used by the Intergovernmental Panel on Climate Change: scenarios are coherent, internally consistent depictions of pathways to possible futures based on assumptions about economic, ecologic, social, political, and technologic development (Nakicenovic et al. 2000). Scenarios generally include qualitative story-lines that describe assumptions about the initial state and the driving forces, events, and actions that lead to future conditions; models that quantify the storyline; outputs that explore possible future outcomes if assumptions are changed; and explicit consideration of uncertainties.

Health scenarios can be constructed by augmenting existing scenarios or by developing new scenarios. The development of new scenarios can start with current conditions and describe how driving forces could result in alternative futures, or it can start with a desirable future and describe what would need to change (and by when) to achieve it. Scenario storylines need to be grounded in an understanding of current conditions and in the factors that shape human health today. Underlying quantitative health scenarios are models of how particular sectors (e.g., technology) may change over time. Health scenarios become policy relevant as they identify and address the questions that are important to decision makers. Scenarios promise to improve our understanding of the effects of climate change and other global changes on current health policies.

## Research Agenda

Efforts are just beginning to explicitly incorporate health into scenario development. To inform this process, the expert panel developed a research agenda in three related areas: development of health models, development of health scenarios, and information and approaches needed to inform related policy issues. The roadmap drawn for each area identifies critical research gaps that will be important to research and funding organizations.

### Development of health models.

Modeling health end points is a complex, multideterminant process that requires attention to an array of factors across dimensions of time and space. Participants concluded that the process of model development should address the following questions:

- What model(s) would be appropriate to incorporate climate change, adaptation measures, and mitigation policies?
- How do we quantify uncertainty?
- How do we validate the model?

Key considerations in health model development include the following:

1. Identifying the key proximal determinants of health and how they can be modeled to project risks and vulnerabilities. In particular, a better understanding is needed of the following:
  - The relationships between climate and health: A better understanding of the associations between climate and health outcomes will aid the development of appropriate adaptive responses to reduce the current and future burden of climate sensitive diseases.
  - The determinants of economic development: Many of the determinants of economic development are also determinants of population health.
  - Indirect relationships and joint effects linking climate with other important global changes in the model: Climate can affect health indirectly through other systems; for example, climate can influence ecosystems in ways that can increase or decrease the intensity of malaria transmission. Climate and land use change can independently and jointly influence the range of some disease vectors, for example, by affecting the intensity and recurrence of droughts. A better understanding is needed of the interrelationships among climate, ecosystems, land use, and health to design effective intervention measures.
  - Scale interactions: Population health is affected by a multitude of factors that operate at different scales, from community (e.g., whether or not there is an effective early warning system for heat waves) to individual levels (e.g., whether or not an individual spends adequate time in cooled environments during a heat wave). Climate change is a global phenomenon whose impacts will primarily be felt at regional and local scales. Local actions, such as land use change that contributes to heating or cooling of the environment, can amplify or ameliorate larger scale climate forces. Methods need to be developed for improving model accuracy by incorporating effects across multiple scales.
  - Methods for downscaling variables such as a country’s gross domestic product to regional and local scales: The mechanics of down-scaling national variables, particularly those that are unevenly distributed across a population, need to be understood.
2. Modeling, on appropriate scales, the processes of adaptation and adaptive capacity. Few models exist of either how to build public health capacity or how the implementation of specific interventions, such as development of a malaria vaccine, is likely to influence population health. Such models are necessary for estimating the potential for adaptation to reduce the projected negative consequences of climate change. Overconfidence in the effectiveness of adaptation can lead to underestimation of the future burden of disease due to climate change. In addition, models are needed of the consequences, including co-benefits, of mitigation policies at appropriate scales.
3. Modeling critical thresholds and nonlinearities. For example, mortality typically exhibits a curvilinear relationship with ambient temperature, with mortality rates increasing with increasing ambient temperature (above the temperature at which mortality is at a minimum) (Kovats and Koppe, in press). In some regions, there is a sharp rise in mortality at very high temperatures. Models are needed to accurately represent these thresholds.
  Health models also need to explore how thresholds or nonlinearities in the climate system may affect population health. Although early climate change projections suggested that global mean surface temperature may gradually increase, there is growing concern that not only will mean temperature and precipitation change, but that changes in the variance around these variables could result in large increases in extreme events in some regions (Albritton and Meira Filho 2001). In addition, the climate record shows evidence of abrupt (on geologic scales) nonlinear changes. Research is needed to better understand the potential consequences of abrupt climate changes for population health.
4. Integrating top-down and bottom-up models. Top-down models are developed from an overall population health perspective that focuses on just one or a few indicators, such as life expectancy. Bottom-up models of population health aggregate individual health outcomes up to the population level. Methods are needed to merge these two approaches to improve our understanding of the key determinants of population health.
5. Validating models and their results. The larger and more complex a model, the more difficult validation becomes, particularly when data are limited. For example, a number of groups have developed models to project how malaria might spread under particular assumptions about changes in temperature and precipitation (e.g., Martens et al. 1999; Rogers and Randolph 2000; Tanser et al. 2003; Van Lieshout et al. 2004). Data for validation of these models are limited. Until more long-term data sets are collected, approaches need to be developed to provide policy makers with confidence that decisions based on model results will be robust.
6. Involving stakeholders, including representatives from multiple disciplines, in model development and validation.
7. Communicating model results, including underlying assumptions and the degree of uncertainty associated with the results, to all interested parties.

### Development of health scenarios.

Many of the issues identified as important for model development are also important for developing scenarios to characterize possible impacts of global change. Health scenarios are particularly sensitive to time scale, level of aggregation, the validity of underlying health models, the degree of confidence in the model, and uncertainties in the scenario.

Given these sensitivities, scenario building should follow a three-step approach. The first step is to determine why the scenario is being built. Reasons generally include strategic planning, risk management, policy setting, and communication. The purpose will determine how the scenario is constructed (impact vs. vulnerability), the scope of the baseline conditions to be incorporated, and the geographic and temporal scale for the scenario. For example, if the scenario is being built to assess possible future vulnerability, then it needs to start with an understanding of regional and population vulnerabilities. For strategic planning and risk management, the scenario should help to identify future needs and priorities for research and adaptation measures to protect human health and well-being. The second step is to determine the kind of scenarios that need to be created to achieve the study purpose. The third is to select the methods and tools appropriate for generating such scenarios. The terminology used should be explicitly defined because different disciplines define the same terms differently (e.g., vulnerability relates to residual damage in natural hazards research and to the current burden of disease in public health). Multiple disciplines should be involved to develop models and scenarios that place climate change in perspective alongside other drivers of health outcomes.

Key considerations in health scenario development include the following:

1. Identifying key determinants of population health and how they will evolve over time. Determinants include climate change, geography (including the built environment), demographics, social behavior, medical and other technology, regional and global economics, and unforeseen events. The degree of predictability of these determinants decreases as time is projected forward, with more certainty in short-term health statistics (e.g., deaths, illnesses, and injuries), less certainty in the medium term (e.g., demographic trends), and even less certainty in long-term determinants of health (including social and economic conditions and genetic change).
2. Incorporating development issues. The environmental, social, economic, technologic, political, and other determinants of population health also are determinants of development. In addition, population health status is both a determinant and a consequence of development. Research is needed to understand how to incorporate the pathways to development in health scenarios.
3. Incorporating adaptation and adaptive capacity into scenarios. Adaptation will determine the difference between the potential and actual impacts of climate change—that is, the strategies, policies, and measures implemented to reduce the projected consequences and take advantage of the opportunities that will arise. Specific adaptations will arise from the adaptive capacity of a population. Research is needed on how to develop storylines that incorporate adaptive capacity and specific adaptation measures over time, together with the consequences on climate-related health impacts. A related issue is how to incorporate the effects of multilateral environmental agreements on vulnerability and adaptation.
4. Incorporating thresholds and nonlinear events into scenarios. Most scenarios assume that change, including climate change, is monotonic. However, ample evidence demonstrates that many systems have thresholds that, when crossed, produce rapid and nonlinear change. These rapid and nonlinear changes, or “surprises,” are a special and extreme sort of uncertainty that need to be explored and integrated into scenarios. Clearly, change of this magnitude and speed could have profound effects on population health.
5. Identifying events or processes that can change projected trends and lead to alternative futures. Uncertainties abound in the relationships among environment, human health, and society, including the constituents and boundary conditions of the problem of concern, the relationships among the system components, the relationships with the external environment, and the future evolution of external forcings.
6. Identifying and characterizing critical uncertainties. There are several sources of uncertainty associated with scenario development, including determining whether the full range of not-improbable futures is captured, ensuring that appropriate models are chosen, and determining whether is it appropriate to assume that associations and assumptions remain constant across geographic and temporal scales. In addition, there is uncertainty in underlying variables (e.g., the rate, speed, and regional extent of climate change; changes in economic development and technology), in response function differences across populations, and in the effectiveness of mitigation and adaptation measures.
7. Involving stakeholders, including decision makers and representatives from a range of pertinent disciplines, in scenario development. Developing scenarios in isolation from users of the scenarios and from other disciplines can result in improbable visions of pathways to the future. Including a range of stakeholders will inform the scenario process and can ensure broad-based support across disciplines.
8. Communicating to stakeholders the scenario process, including the scenario purpose, outcome, and uncertainties. This involves determining which communication methods are most effective for particular stakeholder groups.
9. Evaluating scenarios (including monitoring and mapping of trends).

### Use of health models and scenarios to inform policy.

Carefully developed health scenarios promise to outline plausible health futures from which policy makers can develop reasoned adaptation and mitigation responses. Several issues were identified that address the particular concerns of decision makers, including the following:

1. Demonstrating the value of scenarios to policy makers. Climate change projections suggest that future weather patterns will be different from those experienced today. Making decisions based on current climate could decrease future adaptive capacity. Scenarios provide boundaries within which to test the consequences and robustness of policy choices.
2. Involving decision makers and policy makers in scenario development and eliciting information regarding the questions that are important to them.
3. Determining the temporal and spatial scale needed for decision making. Climate change impacts will be site specific and path dependent. For example, malaria outbreaks occur after the rainy season in some regions but occur during the dry season in other areas. Decision makers need information on the appropriate scale for the development of effective and efficient response measures.
4. Evaluating how current health policies may be affected by a changing climate. Current policies were designed based on recent climate conditions. Some policies will need to be modified with changing weather patterns. For example, lengthening the malaria transmission season may result in the need to treat bed nets more than once per season. Health policies (and health infrastructure) will need to be adjusted to take this change into account.

## Conclusions

We identified several overarching issues that were important to both the process of model and scenario development. For example, it is critical that the purpose of the model or scenario, including the temporal, geographic, and organizational scales within which it will be built, be defined clearly during development. The terminology should be explicit because different disciplines define terms differently. Multiple disciplines should be involved in model and scenario development; in particular, the climate modeling community should be included.

More comprehensive, long-term data sets on finer scales are needed for key determinants of population health to develop models and scenarios that put climate change into perspective with other drivers of health outcomes. Health models also can be improved by better understanding of climate–health associations, better understanding and models of moderating influences (e.g., population growth and level of development), better understanding and models of adaptation measures, quantification of uncertainty, and validation.

Scenario development has moved from narrow, disciplinary-based projections to multidisciplinary integrated approaches; from solely quantitative approaches with simulation models to comprehensive approaches with detailed qualitative narratives; from expert-based approaches to processes characterized by stakeholder involvement; and from a focus on likely futures to a focus on lessons that can be learned. The comprehensive inclusion of health issues in narrative scenarios with the involvement of stakeholders can now be achieved.

The workshop concluded that research on the development of health models and scenarios for global environmental change is of critical importance.

## Figures and Tables

**Table:** 

**Workshop Participants**		
Carlos Corvalan	Maud Huynen	Joel Scheraga
World Health Organization	Maastricht University	U.S. Environmental Protection Agency
Kristie Ebi	Rik Leemans	Jacinthe Sequin
Exponent Health Group	Wageningen University	Health Canada
Paul Epstein	Bettina Menne	Michael Slimak
Harvard Medical School	World Health Organization	U.S. Environmental Protection Agency
Majid Ezzati	Antonio Navarra	Roger Street
Harvard School of Public Health	Instituto Nazionale di Geofisica e Vulcanologia	Meteorological Service of Canada
Janet Gamble	Hugh Pitcher	Brian Thomas
U.S. Environmental Protection Agency	University of Maryland	Swiss Re America
Martha Garrett	John Reilly	Ferenc Toth
Uppsala University	Massachusetts Institute of Technology	International Atomic Energy Agency
Anne Grambsch	Dieter Riedel	Mary Wilson
U.S. Environmental Protection Agency	Health Canada	Harvard School of Public Health
